# The Utility of Lung Ultrasound Scoring in Predicting Post‐Extubation Respiratory Support After Congenital Heart Surgery

**DOI:** 10.1002/jum.16668

**Published:** 2025-03-10

**Authors:** Yordan Hristov Georgiev, Bianca Haase, Felix Neunhoeffer, Johannes Nordmeyer, Ilias Tsiflikas, Jörg Michel, Maximilian Gross

**Affiliations:** ^1^ Department of Pediatric Cardiology, Pulmonology and Intensive Care Medicine University Children's Hospital Tübingen Germany; ^2^ Department of Diagnostic and Interventional Radiology University Hospital Tübingen Tübingen Germany; ^3^ Department of Neonatology University Hospital Tübingen Tübingen Germany

**Keywords:** congenital heart disease, cardiac surgery, lung ultrasound, pediatric intensive care, post extubation strategy

## Abstract

**Objectives:**

Lung ultrasound (LU) is effective in diagnosing the accumulation of extravascular lung water and assessing real‐time fluid status in infants following congenital cardiac surgery with cardiopulmonary bypass. This study evaluated whether LU can be used as a prognostic marker for changes in noninvasive respiratory support after extubation.

**Methods:**

Infants with congenital heart disease (CHD) <1 year of age requiring mechanical ventilation for more than 24 hours postoperatively were included. Using a linear probe, 3 scan fields from each hemithorax were assessed for B‐lines and consolidations, with scores ranging from 0 to 3 assigned per area. LU scores were rated then by 4 independent operators. After extubation, patients were monitored for respiratory support modifications over the following 48 hours and were divided into 3 subgroups: steady state, escalation, and de‐escalation, accordingly.

**Results:**

In this single‐center observational pilot study, a total of 30 patients with a median age of 116 (interquartile range: 17–196) days were included in the prospective analysis between July 2022 and December 2023. LU scores differed significantly among groups: 3.47 ± 2.3 (steady state), 6.14 ± 2.55 (escalation), and 1.63 ± 1.41 (de‐escalation), *P* = .002. ROC analysis identified a cut‐off score of ≥5 as predictive of escalation risk with a sensitivity of 86% and specificity of 83%. A score <2 suggested potential for de‐escalation within 48 hours, with a sensitivity of 75% and specificity of 73%.

**Conclusions:**

LU scoring may be a valuable tool for optimizing ventilator weaning and post‐extubation respiratory strategies in infants undergoing congenital cardiac surgery. Further studies are warranted to validate these findings.

**Abbreviations**
ANOVA: analysis of variance
CHD: congenital heart disease
CPB: cardiopulmonary bypass
EVLW: extravascular lung water
HFNC: high‐flow nasal cannula
IQR: interquartile range
LFNC: low‐flow nasal cannula
LU: lung ultrasound
NIV: non‐invasive ventilation
PEEP: positive end‐expiratory pressure
PICU: pediatric intensive care unit
PIP: peak inspiratory pressure
ROC: receiver operating characteristic

Infants undergoing congenital heart surgery often experience significant pulmonary complications due to the effects of cardiopulmonary bypass (CPB) and aortic clamping on respiratory function.^1^, ^2^, ^3^, ^4^ Intraoperative ischemia and subsequent reperfusion can trigger a systemic hyperinflammatory response, increasing microvascular permeability.^5^ As a result, postoperative issues such as the accumulation of extravascular lung water (EVLW), impaired oxygenation, and pulmonary hypertension frequently occur.^5^

Lung ultrasound (LU) has shown considerable promise in the early postoperative period after pediatric cardiac surgery despite its apparent underutilization in daily care.^2^ It might be used as a complementary tool to conventional chest X‐rays, as it may provide rapid bedside information about the underlying pathological condition in certain situations, for example, pneumothorax and pleural effusion, as well as parenchymal disorders like atelectasis, edema, and pneumonia.^2^, ^6^ LU can be useful in the diagnostic process of EVLW accumulation and assess real‐time fluid status, which is important for postoperative decision‐making and therapy planning.^7^, ^8^ It has been shown to predict the duration of mechanical ventilation and length of stay in the pediatric intensive care unit (PICU) following cardiac surgery.^8^ LU has also been associated with reduced use of ionizing radiation, bedside availability, and the ability to complement traditional markers for risk assessment in pediatric cardiac surgery.^2^, ^9^ Additionally, LU has contributed to reclassifying routine X‐ray findings in this population.^7^

Given LU's ability to detect EVLW accumulation and its association with prolonged mechanical ventilation, we hypothesized that LU could also serve as a prognostic marker for post‐extubation respiratory support needs, potentially optimizing ventilator weaning, and post‐extubation strategies.^8^, ^10^ Specifically, we aimed to investigate whether LU scores could predict the need for modifications in non‐invasive respiratory support within 48 hours after extubation in infants undergoing congenital heart surgery with CPB.

In addition to this primary objective, we also aimed to assess the interrater reliability of LU assessments between pediatric intensive care fellows and radiologists. This pilot study was intended to provide foundational data to guide a larger prospective investigation.

## Materials and Methods

### Study Design, Setting, and Consent

This single‐center observational pilot study was conducted at the 14‐bed tertiary PICU of the University Children's Hospital Tübingen, Germany, between July 2022 and December 2023.

Approval was obtained from the local ethics committee (application no. 200/2022BO). All study procedures followed the *Guidelines for Good Clinical Practice* and ethical standards in the 1964 *Declaration of Helsinki* and its later amendments. Written informed parental consent was obtained upon patient recruitment.

### Patients, Equipment, and Outcome

Infants with congenital heart disease (CHD) under 1 year of age requiring mechanical ventilation for >24 hours after CPB surgery were included. Exclusion criteria were length of PICU stay <24 hours, severe thoracic deformities, or coexisting pulmonary conditions (eg, bronchopulmonary dysplasia, lung hypoplasia, pulmonary sequestration).

Postoperative ventilation was carried out using a constant flow ventilator (Leonie plus, Löwenstein medical, Bad Ems, Germany). Infants were ventilated using synchronized intermittent mandatory ventilation or synchronized intermittent positive pressure ventilation combined with pressure support, adjusted according to clinical needs. At least 12 hours prior to extubation, ventilator settings were standardized to a positive end‐expiratory pressure (PEEP) of 5 mbar, peak inspiratory pressure (PIP) <20 mbar, and FiO_2_ <0.4, targeting predefined oxygen saturation levels. Extubation decisions were made during morning rounds, deciding on post‐extubation respiratory support according to the infant's clinical condition, ventilation status, and chest X‐ray. Procedures were completed within 4 hours, aligning with the nursing shift schedule.

Infants were observed for 48 hours post‐extubation and grouped as follows: group 1—steady state, without changes in respiratory support; group 2—escalation, increase in respiratory support, defined as transitioning from low‐flow nasal cannula (LFNC) or no support to high‐flow nasal cannula (HFNC) or non‐invasive ventilation (NIV), HFNC to NIV, or HFNC/NIV to re‐intubation; and group 3—de‐escalation, reduced respiratory support, defined as transitioning from NIV to HFNC, HFNC to LFNC, or LFNC to no support. HFNC was applied with a flow of 2L/kg body weight per minute using an HFNC device with the respective patient interface (AIRVO 2 and Optiflow Junior Nasal Cannula‐Infant, Fisher & Paykel Healthcare Limited, Auckland, New Zealand). NIV was applied using binasal prongs (Fitz Stephan GmbH, Gackenbach, Germany) and a constant flow ventilator (Leonie plus, Löwenstein Medical, Bad Ems, Germany) with a PEEP of 5 mbar and a PIP of 14–16 mbar.

### Lung Ultrasound Scoring (LUS)

Two experienced examiners (Y.G. and M.G.) performed the LU within 4 hours before extubation. LU was performed using a Mindray L14‐5W Linear Array Probe and a Zonare Z One Pro (Zonare Medical Systems, California, USA) ultrasound device. The probe was positioned parallel to the sternum, covering 3 scan fields from each hemithorax: upper frontal, lower frontal, and lateral subaxillary. Six 3‐second videos were recorded for each patient. Two experienced sonographers, a pediatric radiologist and a neonatologist in the second year of pediatric radiology training (B.H. and I.T.) and 2 pediatric intensive care fellows (Y.G. and M.G.), blinded to all patient data, evaluated the findings and scored them according to the 4‐point scale.^11^ Zero points were given if there were A‐lines without B‐lines or isolated B‐lines with still preserved A‐lines. One point was given if single B‐lines were present without A‐lines in 1 intercostal space. We gave 2 points if there were multiple well‐spaced B‐lines in the entire scanned field or coalescent B‐lines, and 3 points if there were lung consolidations with or without pleural effusion. Example videos are included in the Supporting Information. We summed the scores of all scanned fields in both lungs, so that each patient ultimately received a single score.

### Statistical Analysis

Statistical analyses were performed using SigmaPlot (Version 13 for Windows, Systat Software, Inc., San Jose, CA, USA) and Statistical Package for Social Sciences Version 29 (IBM Corp., Armonk, NY, USA). A convenience sample size of 30 patients was selected for our pilot study to assess the feasibility and initial outcomes. Continuous data are presented as median and interquartile range (IQR) and categorical data are presented as frequencies and percentages. Comparisons of 2 or more groups were performed using 1 way analysis of variance (ANOVA). All *P*‐values <.05 were considered statistically significant. Data were tested for normality using the Shapiro–Wilk test. If the test failed, results were provided as median and IQR; if passed, the results were given as mean and standard deviation. The diagnostic accuracy of the LU was investigated using the receiver operating characteristic (ROC) for both the increase and decrease of post‐extubation respiratory support. To assess diagnostic performance, the Youden index was applied, optimizing the balance between sensitivity and specificity for threshold determination. Intraclass correlation was used to evaluate interrater agreement.^12^

## Results

During the investigated period between July 2022 and December 2023, 180 patients under 1 year of age underwent surgery in our center. After excluding patients who were operated on without CPB, were extubated on the first postoperative day, or had pre‐existing pulmonary disease or prematurity, 30 patients were included in the study. They were divided into 3 subgroups based on the changes in post‐extubation respiratory support strategies as stated above (Table 1).

**Table 1:** Patients Characteristics and Descriptive Statistics of the 3 Subgroups

	All, n = 30	Group 1 Steady State, n = 15	Group 2 Increase in Respiratory Support, n = 7	Group 3 Decrease in Respiratory Support, n = 8	*P*‐value
Age (days) (IQR)	116 (17–196)	112 (53–141)	193 (12–214)	86.5 (17.3–165)	.957
Weight (kg) (IQR)	4.6 (3.52–5.59)	4.70 (3.94–5.55)	4.5 (2.8–6.51)	4.35 (3.35–5.32)	.757
CPB time (min) (IQR)	120.5 (86.8–140.5)	114.3 ± 63.4	121.7 ± 35.7	129.1 ± 43.2	.814
Cross‐clamp time (min) (IQR)	70 (43.8–109.3)	75.9 ± 50.7	66.3 ± 52.1	88 ± 55.3	.724
Palliation, n (%)	11 (37)	4 (27)	4 (57)	3 (37)	.397
Length of hospital stay (days) (IQR)	27 (16.3–53.3)	29 (15–58)	22 (22–56)	20.5 (13–30.5)	.464
Length of PICU stay (days) (IQR)	11 (6–20.5)	12 (6–20)	12 (8–24)	7 (4.5–19)	.366
Duration of mechanical ventilation for extubation (days) (IQR)	4 (2–9.5)	8 (3–13)	3 (1–4)	3 (2.3–6.5)	.045*
LUS, mean ± SD	3.6 ± 2.65	3.47 ± 2.3	6.14 ± 2.55	1.63 ± 1.41	.**002** *

The data regarding CPB time, cross‐clamp time, and LUS demonstrated normal distribution and are represented as mean and standard deviation. The rest of the data were not normally distributed and are accordingly represented as median and interquartile range. CPB, cardiopulmonary bypass; LUS, lung ultrasound score; PICU, pediatric intensive care unit.

* All *P*‐values < .05 were considered statistically significant.

The 3 subgroups demonstrated homogeneity regarding age, weight, operation strategy, CPB time, and cross‐clamp time. No statistically significant differences were observed in the length of hospital stay or PICU stay among subgroups. However, the patients in the de‐escalation group had a shorter PICU stay than the steady state and escalation groups (7 versus 12 versus 12 days). Additionally, the duration of mechanical ventilation prior to extubation differed significantly between the steady state and the escalation group (8 versus 3 days, p = 0.045).

Interestingly, the infants in the steady‐state and de‐escalation groups more frequently received NIV after extubation compared to the escalation group—6/15 (40%) and 4/8 (50%) versus 1/7 (14%), respectively.

**Figure 1:**
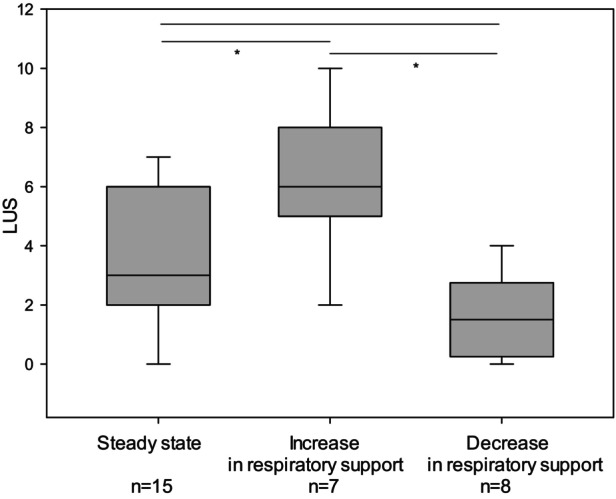
Boxplot of LUS in the 3 subgroups; the Shapiro–Wilk test revealed normal distribution (*P* = .615), n = 30. Analysis of variance showed statistically significant differences—steady state group versus escalation group *P* = .023, escalation versus de‐escalation group *P* = .001 (bars with *). Between the steady state and de‐escalation groups, there was no statistically significant difference—*P* = .062. LUS, lung ultrasound score.

LU findings also varied significantly among the 3 subgroups, with mean LU scores as follows: steady state versus escalation versus de‐escalation group 3.47 ± 2.3 versus 6.14 ± 2.55 versus 1.63 ± 1.41, *P* = .002, respectively (Table 1 and Figure 1).

The interrater reliability for the LU scoring in all 180 LU‐scanned zones was high, with an intraclass correlation coefficient of 0.85 (95% CI 0.74–0.92; *P* < .01).

**Figure 2:**
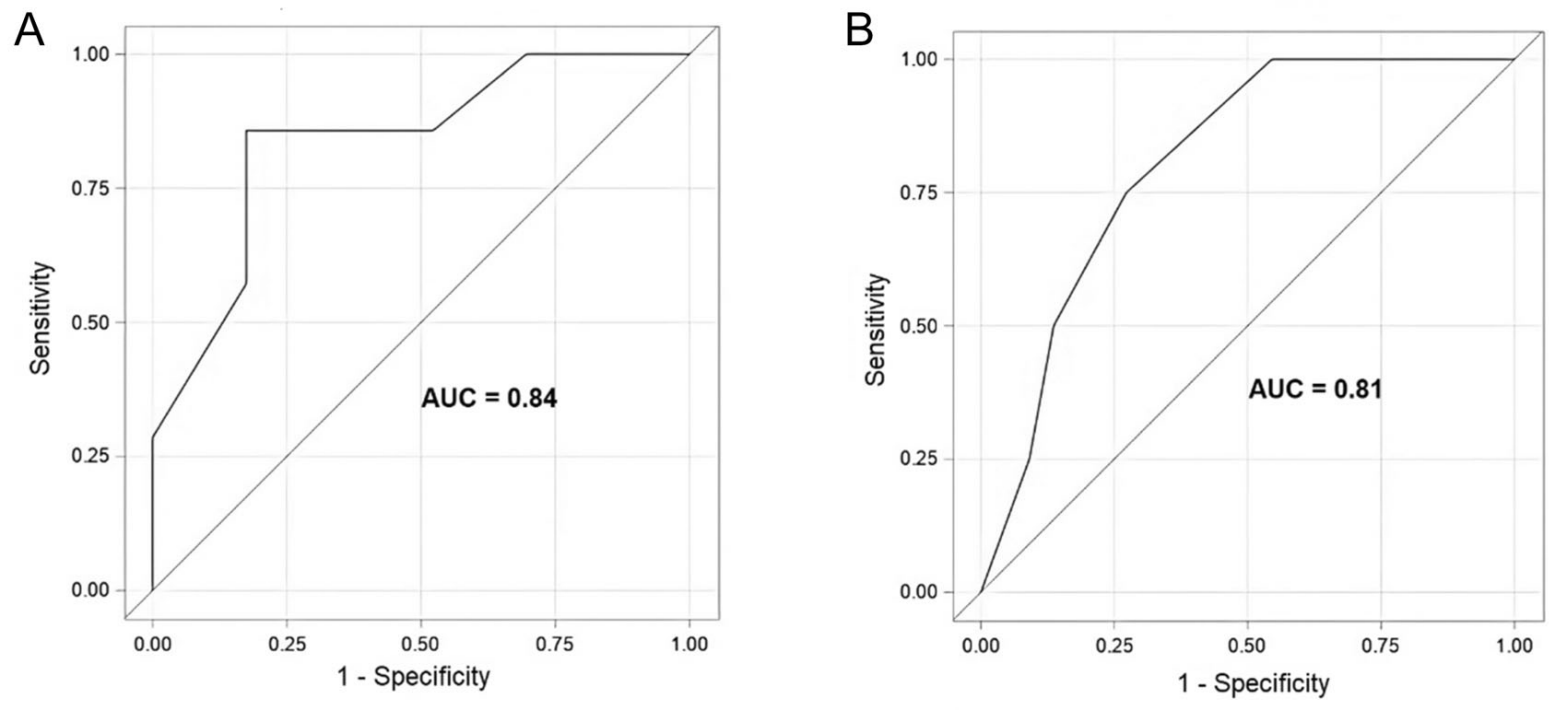
**A**, ROC curve demonstrating the sensitivity and specificity of LU to predict the increase in respiratory support within 48 hours following extubation. **B**, ROC curve demonstrating the sensitivity and specificity of LU to predict the decrease in respiratory support within 48 hours following extubation. ROC, receiver operating characteristic curve.

The diagnostic accuracy of LU for predicting increased respiratory support within 48 hours following extubation was established using a ROC plot (Figure 2A). A 1‐point increase in LUS corresponded to a 1.83‐fold greater risk of escalating respiratory support after extubation (95% CI 1.13–2.97, *P* = .01). The Youden index reached its highest value at a LUS of 4.99, with a sensitivity of 86% and specificity of 83%. Dichotomizing the LUS at <5 versus ≥5 indicated a higher risk for children with a LUS of ≥5 for escalating respiratory support after extubation, with an odds ratio of 28.5 (95% CI 2.65–306.59, *P* = .01).

Similarly, the diagnostic accuracy of LU for predicting decreased respiratory support within 48 hours following extubation was established using a ROC plot (Figure 2B). A 1‐point decrease in LUS corresponded to a 0.55‐fold lower risk of escalating respiratory support after extubation (95% CI 0.32–0.93, *P* = .03). The Youden index reached its highest value at a LUS of 1.99, with a sensitivity of 75% and specificity of 73%. Dichotomizing LUS at ≥2 versus <2 indicated a higher probability for children with a LUS of <2 for de‐escalating respiratory support within 48 hours after extubation, with an odds ratio of 6.33 (95% CI 1.00–40.07, *P* = .05).

## Discussion

We conducted a prospective observational pilot study to assess the utility of LU in predicting post‐extubation respiratory support in infants who underwent congenital heart surgery with CPB.

In our study, as expected, LUS were the lowest in the de‐escalation group and the highest in the escalation group. While the steady‐state group demonstrated significantly lower scores than the escalation group, their scores were closer to those of the de‐escalation group. This aligns with previous studies suggesting that elevated LU scoring is associated with worse respiratory outcomes.^8^, ^10^

Several previous studies reported LU's utility in visualizing pulmonary effects and predicting clinical outcomes in pediatric cardiac surgery patients.^2^, ^6^, ^8^, ^9^, ^13^ However, few studies focused explicitly on assessing post‐extubation respiratory trajectories in this population, limiting direct comparison to our findings. A recent study by Hubara et al.^14^ examined the predictive capability of pre‐extubation LU scoring for extubation failure, finding an association between higher LUS and re‐intubation or unplanned need for non‐invasive respiratory support. Given the study's high interobserver variability, which limits its reliability, the authors conclude that LU might be helpful in estimating respiratory trajectories in infants after cardiac surgery, but further studies and consistent training are needed. Gregorio‐Hernández et al. demonstrated that LU within the first 24 hours after cardiac surgery or catheterization could predict the length of hospital stay and respiratory support; however, their study focused on a highly heterogeneous population of neonates, limiting direct comparability to our findings.^15^

Our findings suggest that patients with a LU score ≥5 may benefit from proactive escalated respiratory support regarding post‐extubation strategy to reduce the risk of adverse outcomes.

Integrating clinical and paraclinical data, including ventilation status, imaging, fluid balance, type and duration of surgery, and bypass time, could enhance decision making for post‐extubation strategies.

For patients with a LU score <2, early de‐escalation of respiratory support might contribute to better planning of patient transfers to post‐intensive care settings. As tertiary PICUs might be affected by personnel shortages,^16^ this might be beneficial for managing intensive care beds.

Patients in the steady state group had a longer duration of mechanical ventilation than the other groups and tended to have a longer overall hospital and PICU stay, comparable to the duration observed in the escalation group. We applied the same weaning criteria to all groups before extubation as a standardized institutional approach. However, infants with prolonged mechanical ventilation were more likely to receive non‐invasive respiratory support post‐extubation. As a result, although infants in the steady state group did not need escalation, they experienced longer hospital and PICU stays.

To reduce the duration of invasive mechanical ventilation, daily reevaluation of the weaning process, extubation readiness tests, and early use of non‐invasive respiratory support might be beneficial.^17^ In this context, utilizing LU for better planning of extubation and the post‐extubation respiratory support strategy could be beneficial.

The results of the LU examinations during our study were not presented to the respective PICU team to explicitly avoid influencing the post‐extubation strategy. The pediatric intensivists who performed the LU were not involved in the decision‐making process regarding the post‐extubation strategy. LU analyzing and scoring were carried out after the end of recruitment using pseudonymized videos to avoid biasing the raters' evaluation by the respective clinical course. The LU scoring interrater agreement among our 4 raters was good, suggesting method robustness when used by different practitioners.^18^

We opted for longitudinal (ie, parallel to the sternum) placement of the ultrasound probe, which is common practice in pediatric LU. This allowed us to examine a large area of the lung, including the apical regions, and facilitated proper probe placement despite prominent dressings after median sternotomy.^11^, ^15^

As anterior scan zones have been proven informative concerning respiratory outcomes, we selected anterior and lateral scan zones for LU scoring.^9^, ^19^ In doing so, we acknowledged a potential loss of information, as findings in posterior lung fields, such as consolidations, correlate with poorer outcomes.^2^, ^15^ Following our institution's approach of placing infants supine postoperatively, with limited opportunity for mobilization, posterior scans were deemed impractical during the study's setting. Since the infants were awake and particularly sensitive to extensive movements during ventilator weaning, the scanning protocol with 3 scanning zones for each lung was considered optimal for this population.

Dietrich et al.^19^ discussed the role of different probes and device settings depending on the goal of the analysis in LU studies. For our study, we chose a high‐frequency linear probe. Although the authors question the significance of different transducers in the evaluation of B‐lines and whether this has any practical importance in monitoring pulmonary congestion and aeration, this probe might be more suitable for assessing focal pleural irregularities and B‐line artifacts, particularly in children with a thin chest wall.

## Limitations

Due to its design as a pilot study, the small sample size, chosen primarily for practical reasons, limits the interpretation of our results. Recruitment was restricted to times when the 2 main investigators (Y.G. and M.G.) were available. The relatively wide time frame for LU examinations of up to 4 hours before the actual extubation may also have influenced the results. As there are several studies on children undergoing congenital cardiac surgery, each of them used a different protocol depending on the specific goals. The lack of standardization in LU makes it difficult to compare results across studies and adds an additional limitation.

## Conclusion

LU scoring, as demonstrated in this pilot study, offers a reliable, non‐invasive tool for predicting post‐extubation respiratory support needs in infants after congenital heart surgery. Our findings suggest that LUS of ≥5 indicates a need for respiratory support escalation, while scores <2 suggest readiness for de‐escalation. Further studies are needed to confirm these findings and assess their broader applicability.

## Supporting information


**Video S1.** Zero points: lung ultrasound scoring system; 0 points: well‐differentiated A‐lines.

**Video S2.** One point: lung ultrasound scoring system; 1 point: well‐spaced single B‐lines without A‐lines in 1 intercostal space.

**Video S3.** Two points: lung ultrasound scoring system; 2 points: multiple well‐spaced B‐lines in the entire scanned field.

**Video S4.** Three points: lung ultrasound scoring system; 3 points: lung consolidations with pleural effusion.

## Data Availability

The data that support the findings of this study are available on request from the corresponding author. The data are not publicly available due to privacy or ethical restrictions.
